# Supplementary material for: NNAlign_MA; MHC Peptidome Deconvolution for Accurate MHC Binding Motif Characterization and Improved T-cell Epitope Predictions
Source: Mol Cell Proteomics. 2019 Oct 2;18(12):2459–77. doi: 10.1074/mcp.TIR119.001658 (PMC6885703; doi:10.1074/mcp.TIR119.001658)
Supplement: Supplementary Material [file 154080_2_supp_401205_pybl4c.pdf]

Supplementary Figure 1. Full NNAlign_MA motif deconvolution for the Multi Allele (MA) HLA-I data analyzed in this work.

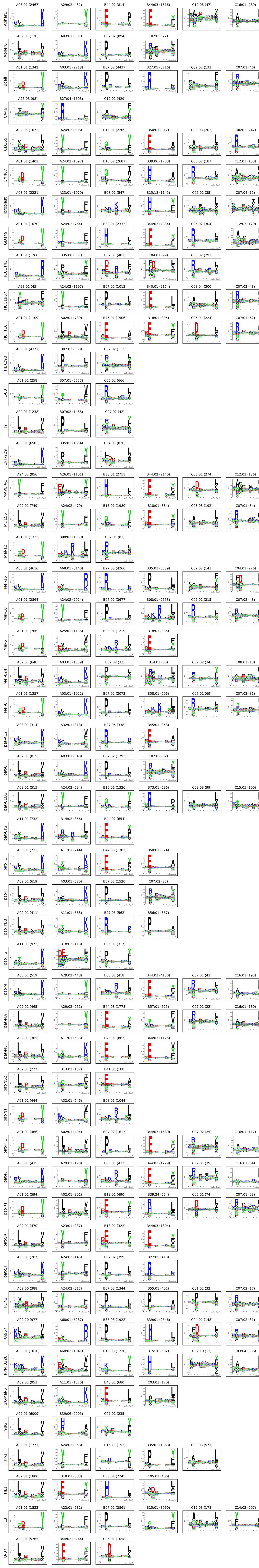

Each row corresponds to a cell line present in the training data (50 in total; for more details, refer to Supplementary Table 4). Using cross validation, each ligand is assigned to one of the HLA alleles expressed in the given cell line. Using this assignment, binding motifs were generated for each allele in each cell line using Seq2Logo (60). To remove potential MS contaminants, only ligands with a prediction score greater than 0.01 were included. Above each logo is given the number of sequences associated to the corresponding HLA allele. For details on the accuracy of this clustering, refer to Supplementary Figures 2 and 3.

Supplementary Figure 2. Comparison between NNAlign_MA deconvoluted motifs and motifs derived from single-allele (SA) data from the IEDB (33) for alleles characterised by at least 100 ligands for both SA data and MA deconvolution data.

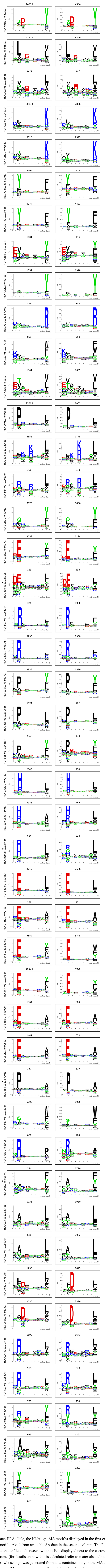

For each HLA allele, the NNAlign_MA motif is displayed in the first column; the motif derived from available SA data in the second column. The Pearson correlation coefficient between two motifs is displayed next to the corresponding HLA name (for details on how this is calculated refer to materials and methods). Alleles whose logo was generated from data contained only in the MA training set (this is, no SA data was present in the training phase) are tagged with an asterisk. To remove potential MS contaminants, only ligands with a prediction score greater than 0.01 were included. The amount of sequences employed to construct a given logo is displayed on top of each logo.

Supplementary Figure 3. Correlation matrices between NNAlign_MA motifs for HLA alleles that are shared between five or more cell lines.

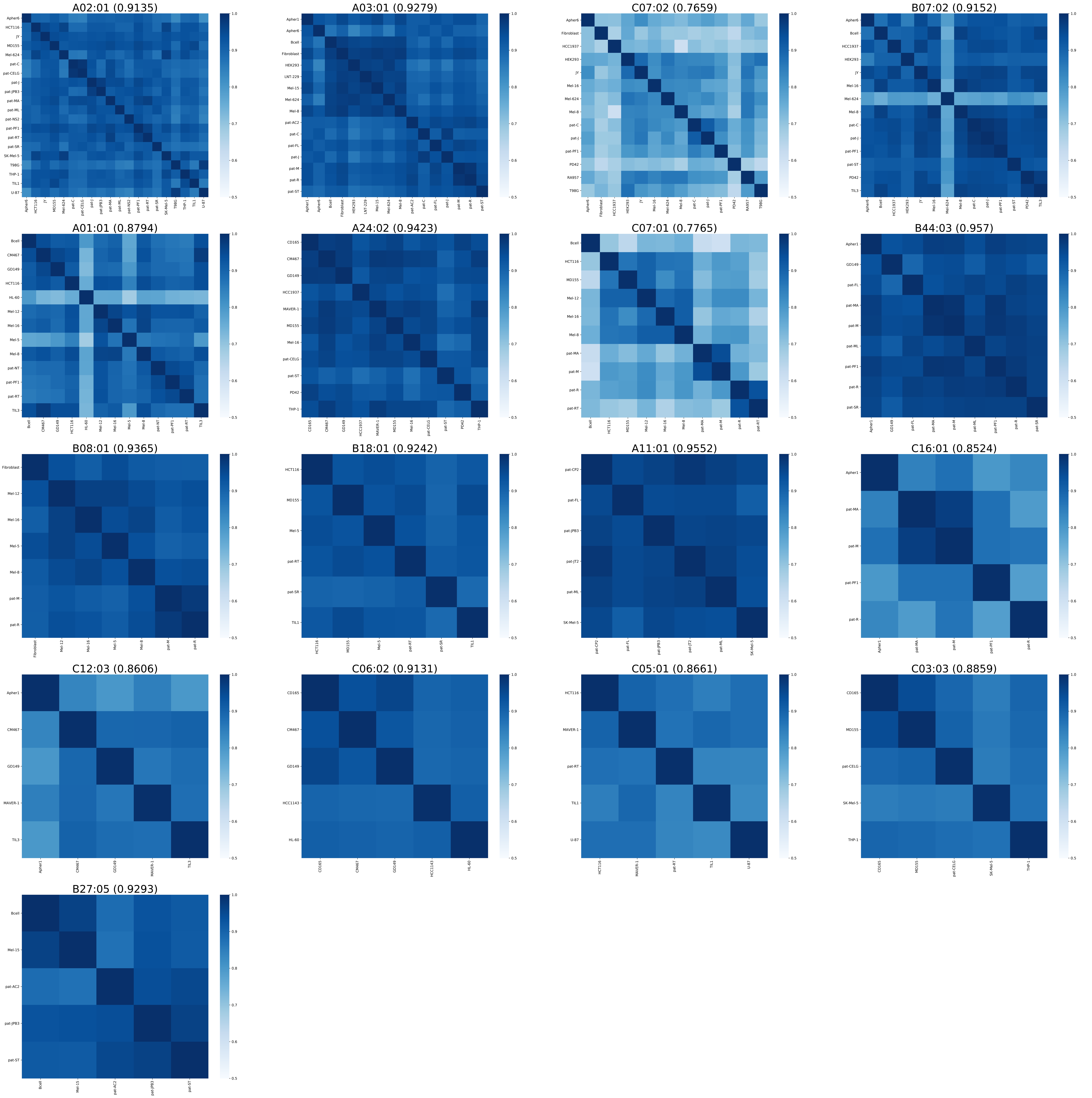

Each matrix displays the Pearson correlation coefficients between all motifs found by NNAlign_MA for a given HLA allele, across all the cell lines sharing the allele (for details on how the correlation is calculated refer to materials and methods). The average Pearson correlation coefficient for each matrix is given next to the corresponding allele name.

Supplementary Figure 4. Full NNAlign_MA motif deconvolution for the Multi Allele (MA) BoLA-I data analyzed in this work.

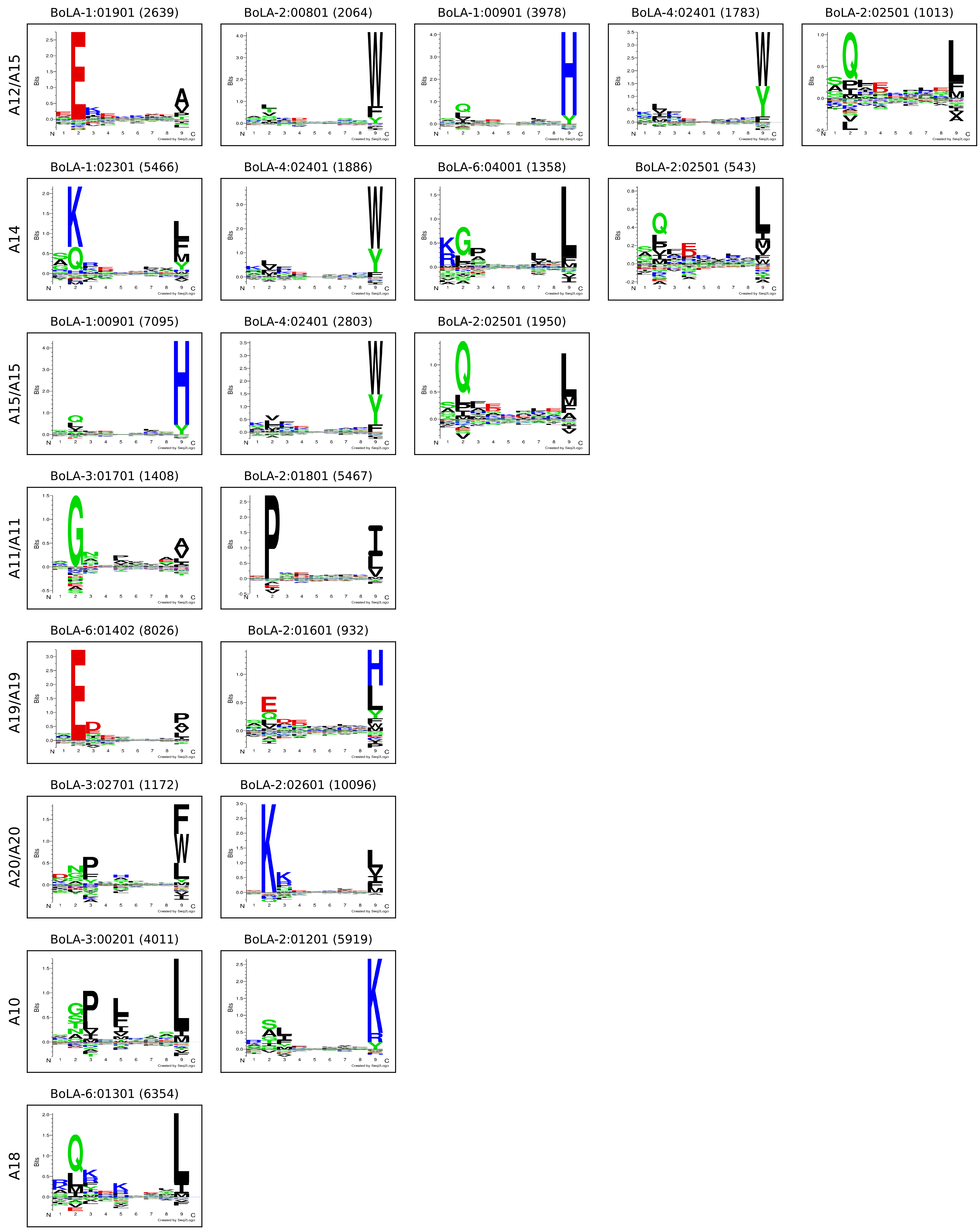

Each row corresponds to a MA data set present in the training data. Using cross validation, each ligand is assigned to one of the BoLA alleles expressed in the given data set. Using this assignment, binding motifs were generated for each allele in each cell line using Seq2Logo (60). To remove potential MS contaminants, only ligands with a prediction score greater than 0.01 were included. Above each logo is given the number of sequences associated to the corresponding BoLA allele.

Supplementary Figure 5. Full NNAlign_MA motif deconvolution for the Multi Allele (MA) HLA-II data analyzed in this work.

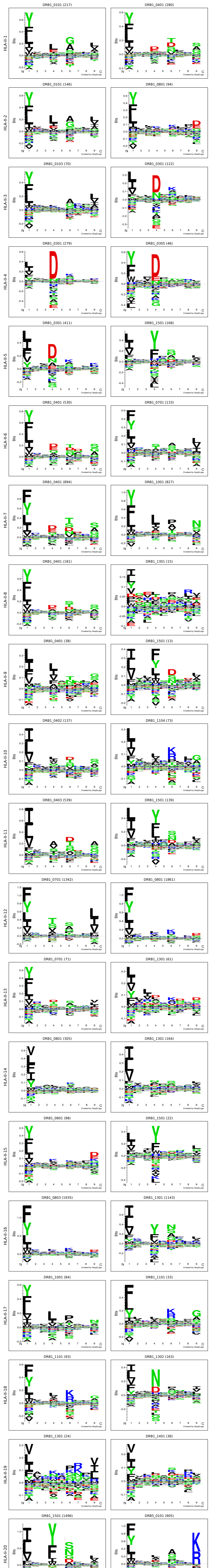

Each row corresponds to a MA data set present in the training data. Using cross validation, each ligand is assigned to one of the HLA alleles expressed in the given data set. Using this assignment, binding motifs were generated for each allele in each cell line using Seq2Logo (60). To remove potential MS contaminants, only ligands with a prediction score greater than 0.01 were included. Above each logo is given the number of sequences associated to the corresponding HLA allele.

Supplementary Figure 6. Prediction score rescaling for the BoLA-2*02501 and BoLA-4*02401 molecules.

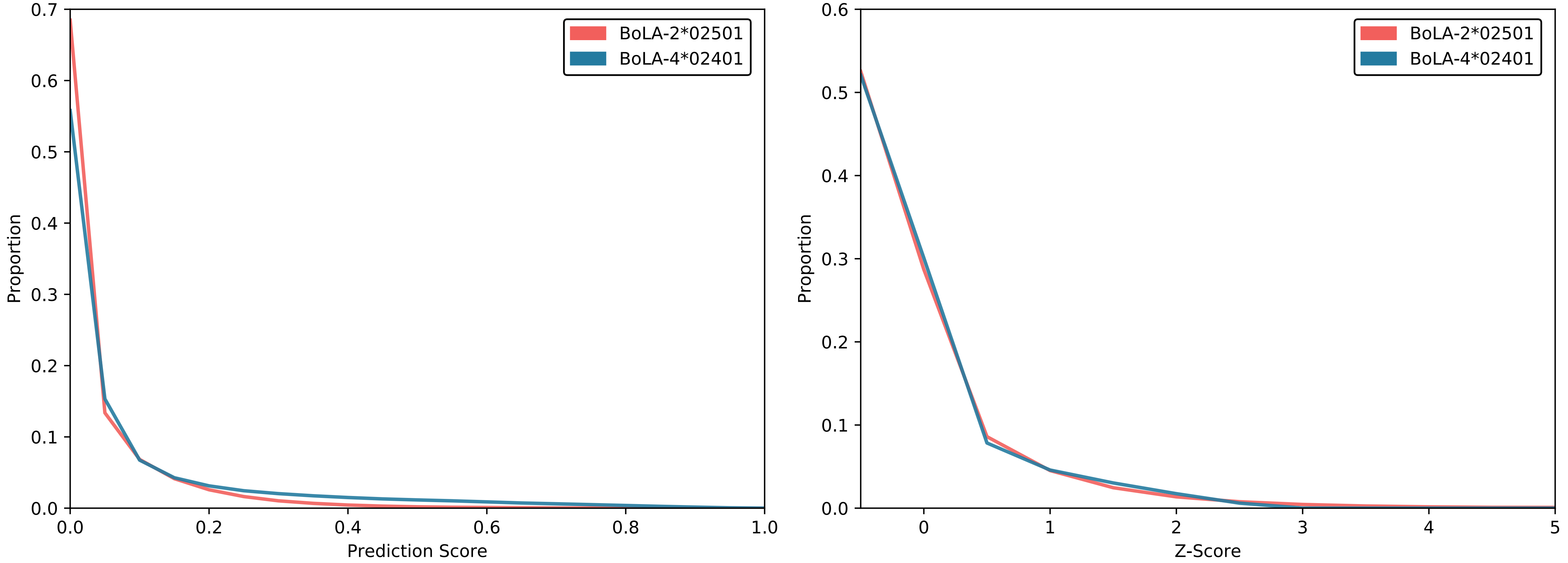

Left panel: proportion of ligands as a function of the prediction scores for both BoLA molecules. In this case, distributions exhibit different characteristics (i.e. more than 13% of random natural peptides have a prediction score greater than 0.3 for BoLA-4*02401, while for BoLA-2*0251 this number is less than 3%). Right panel: proportion of ligands as a function of the Z-Score transformation of the prediction scores. This type of rescaling allows reshaping both distributions so they display similar silhouettes.

Supplementary Table 1. Summary of the multi-allele (MA) data included in the HLA-I benchmark.

Cell line ID	Positives	Negatives	HLA-A		HLA-B		HLA-C		Source PMID
CD165	5364	132869	HLA-A*02:05	HLA-A*24:02	HLA-B*15:01	HLA-B*50:01	HLA-C*03:03	HLA-C*06:02	
CM467	7401	184646	HLA-A*01:01	HLA-A*24:02	HLA-B*13:02	HLA-B*39:06	HLA-C*06:02	HLA-C*12:03	
GD149	9756	208444	HLA-A*01:01	HLA-A*24:02	HLA-B*38:01	HLA-B*44:03	HLA-C*06:02	HLA-C*12:03	
MD155	4374	108036	HLA-A*02:01	HLA-A*24:02	HLA-B*15:01	HLA-B*18:01	HLA-C*03:03	HLA-C*07:01	
PD42	2577	48693	HLA-A*02:06	HLA-A*24:02	HLA-B*07:02	HLA-B*55:01	HLA-C*01:02	HLA-C*07:02	
RA957	11037	232658	HLA-A*02:20	HLA-A*68:01	HLA-B*35:03	HLA-B*39:01	HLA-C*04:01	HLA-C*07:02	28832583
TIL1	5445	140312	HLA-A*02:01	HLA-A*02:01	HLA-B*18:01	HLA-B*38:01	HLA-C*05:01	-	
TIL3	8799	206212	HLA-A*01:01	HLA-A*23:01	HLA-B*07:02	HLA-B*15:01	HLA-C*12:03	HLA-C*14:02	
Apher1	6145	123349	HLA-A*03:01	HLA-A*29:02	HLA-B*44:02	HLA-B*44:03	HLA-C*12:03	HLA-C*16:01	
Apher6	1962	39798	HLA-A*02:01	HLA-A*03:01	HLA-B*07:02	-	HLA-C*07:02	-	
Mel-15	21813	395324	HLA-A*03:01	HLA-A*68:01	HLA-B*27:05	HLA-B*35:03	HLA-C*02:02	HLA-C*04:01	
Mel-16	11980	264233	HLA-A*01:01	HLA-A*24:02	HLA-B*07:02	HLA-B*08:01	HLA-C*07:01	HLA-C*07:02	
Mel-12	3758	88425	HLA-A*01:01	HLA-A*01:01	HLA-B*08:01	-	HLA-C*07:01	-	27869121
Mel-8	6251	119000	HLA-A*01:01	HLA-A*03:01	HLA-B*07:02	HLA-B*08:01	HLA-C*07:01	HLA-C*07:02	
Mel-5	4749	106896	HLA-A*01:01	HLA-A*25:01	HLA-B*08:01	HLA-B*18:01	-	-	
Fibroblast	5289	122127	HLA-A*03:01	HLA-A*23:01	HLA-B*08:01	HLA-B*15:18	HLA-C*07:02	HLA-C*07:04	
HCC1143	2780	69565	HLA-A*31:01	-	HLA-B*35:08	HLA-B*37:01	HLA-C*04:01	HLA-C*06:02	
HCC1937	4976	102331	HLA-A*23:01	HLA-A*24:02	HLA-B*07:02	HLA-B*40:01	HLA-C*03:04	HLA-C*07:02	25576301
HCT116	4174	93208	HLA-A*01:01	HLA-A*02:01	HLA-B*45:01	HLA-B*18:01	HLA-C*05:01	HLA-C*07:01	
JY	2868	60863	HLA-A*02:01	-	HLA-B*07:02	-	HLA-C*07:02	-	
Bcell	12199	220971	HLA-A*01:01	HLA-A*03:01	HLA-B*07:02	HLA-B*27:05	HLA-C*02:02	HLA-C*07:01	24616531
Mel-624	2375	49050	HLA-A*02:01	HLA-A*03:01	HLA-B*07:02	HLA-B*14:01	HLA-C*07:02	HLA-C*08:01	27600516
SK-Mel-5	3293	64537	HLA-A*02:01	HLA-A*11:01	HLA-B*40:01	-	HLA-C*03:03	-	
HEK293	4972	86634	HLA-A*03:01	-	HLA-B*07:02	-	HLA-C*07:02	-	
MAVER-1	7403	171783	HLA-A*24:02	HLA-A*26:01	HLA-B*38:01	HLA-B*44:02	HLA-C*05:01	HLA-C*12:03	
HL-60	6607	115694	HLA-A*01:01	-	HLA-B*57:01	-	HLA-C*06:02	-	
RPMI8226	4524	113201	HLA-A*30:01	HLA-A*68:02	HLA-B*15:03	HLA-B*15:10	HLA-C*02:10	HLA-C*03:04	26992070
THP-1	5542	142866	HLA-A*02:01	HLA-A*24:02	HLA-B*15:11	HLA-B*35:01	HLA-C*03:03	-	
CA46	2324	62647	HLA-A*26:03	-	HLA-B*27:04	-	HLA-C*12:02	-	
LNT-229	10311	177908	HLA-A*03:01	-	HLA-B*35:01	-	HLA-C*04:01	-	
T98G	10011	216072	HLA-A*02:01	-	HLA-B*39:06	-	HLA-C*07:02	-	27412690
U-87	11585	241396	HLA-A*02:01	-	HLA-B*44:02	-	HLA-C*05:01	-	
pat-AC2	1369	32168	HLA-A*03:01	HLA-A*32:01	HLA-B*27:05	HLA-B*45:01	-	-	
pat-C	2983	49759	HLA-A*02:01	HLA-A*03:01	HLA-B*07:02	-	HLA-C*07:02	-	
pat-CELG	3814	72328	HLA-A*02:01	HLA-A*24:02	HLA-B*15:01	HLA-B*73:01	HLA-C*03:03	HLA-C*15:05	
pat-CP2	1790	36895	HLA-A*11:01	-	HLA-B*14:02	HLA-B*44:02	-	-	
pat-FL	3629	74392	HLA-A*03:01	HLA-A*11:01	HLA-B*44:03	HLA-B*50:01	-	-	
pat-J	2552	42497	HLA-A*02:01	HLA-A*03:01	HLA-B*07:02	-	HLA-C*07:02	-	
pat-JPB3	1937	35295	HLA-A*02:01	HLA-A*11:01	HLA-B*27:05	HLA-B*56:01	-	-	
pat-JT2	1467	29587	HLA-A*11:01	-	HLA-B*18:03	HLA-B*35:01	-	-	
pat-M	2476	53262	HLA-A*03:01	HLA-A*29:02	HLA-B*08:01	HLA-B*44:03	HLA-C*07:01	HLA-C*16:01	27841757
pat-MA	3682	69891	HLA-A*02:01	HLA-A*29:02	HLA-B*44:03	HLA-B*57:01	HLA-C*07:01	HLA-C*16:01	
pat-ML	3139	55262	HLA-A*02:01	HLA-A*11:01	HLA-B*40:01	HLA-B*44:03	-	-	
pat-NS2	636	15212	HLA-A*02:01	-	HLA-B*13:02	HLA-B*41:01	-	-	
pat-NT	2190	53238	HLA-A*01:01	HLA-A*32:01	HLA-B*08:01	-	-	-	
pat-PF1	4646	86859	HLA-A*01:01	HLA-A*02:01	HLA-B*07:02	HLA-B*44:03	HLA-C*07:02	HLA-C*16:01	
pat-R	2372	49169	HLA-A*03:01	HLA-A*29:02	HLA-B*08:01	HLA-B*44:03	HLA-C*07:01	HLA-C*16:01	
pat-RT	2537	49846	HLA-A*01:01	HLA-A*02:01	HLA-B*18:01	HLA-B*39:24	HLA-C*05:01	HLA-C*07:01	
pat-SR	2632	57417	HLA-A*02:01	HLA-A*23:01	HLA-B*18:01	HLA-B*44:03	-	-	
pat-ST	1256	26963	HLA-A*03:01	HLA-A*24:02	HLA-B*07:02	HLA-B*27:05	-	-	

“Positives” and “Negatives” refer to the number of positive and negative instances contained in each cell line data. Further rows show the HLA-A, HLA-B and HLA-C expressed by a given cell line, together with the Source ID for its corresponding dataset.

Supplementary Table 2. Single Allele (SA) Binding Affinity (BA) and Eluted Ligands (EL) training data summary for the HLA-I system. For the BA training set, the total amount of sequences per MHC molecule (discarding artificial negatives) is shown; in the case of EL data, the total amount of positives is displayed.

BA data	
**Alleles**	**# Peptides**
HLA-A01:01	3985
HLA-A02:01	11097
HLA-A02:02	3613
HLA-A02:03	5728
HLA-A02:04	3
HLA-A02:05	66
HLA-A02:06	4802
HLA-A02:07	66
HLA-A02:10	18
HLA-A02:11	1081
HLA-A02:12	1181
HLA-A02:16	919
HLA-A02:17	341
HLA-A02:19	1243
HLA-A02:50	134
HLA-A03:01	6513
HLA-A03:02	26
HLA-A03:19	30
HLA-A11:01	5614
HLA-A11:02	14
HLA-A23:01	1871
HLA-A24:02	2315
HLA-A24:03	1374
HLA-A25:01	959
HLA-A26:01	3729
HLA-A26:02	641
HLA-A26:03	535
HLA-A29:02	2286
HLA-A30:01	2711
HLA-A30:02	1564
HLA-A31:01	5152
HLA-A32:01	908
HLA-A32:07	88
HLA-A32:15	74
HLA-A33:01	2508
HLA-A66:01	207
HLA-A68:01	3190
HLA-A68:02	4542
HLA-A68:23	82
HLA-A69:01	2559
HLA-A74:01	15
HLA-A80:01	1167
HLA-B07:02	4100
HLA-B08:01	3171

EL data	
**Alleles**	**# Peptides**
HLA-A01:01	3405
HLA-A02:01	5349
HLA-A02:05	98
HLA-A02:07	30
HLA-A03:01	427
HLA-A11:01	313
HLA-A24:02	2699
HLA-A24:06	169
HLA-A24:13	49
HLA-A26:01	97
HLA-A29:02	4377
HLA-A31:01	31
HLA-A32:01	29
HLA-B07:02	3324
HLA-B08:01	458
HLA-B13:01	57
HLA-B15:01	455
HLA-B15:02	52
HLA-B18:01	46
HLA-B27:02	2334
HLA-B27:03	278
HLA-B27:04	569
HLA-B27:05	2567
HLA-B27:06	646
HLA-B27:07	1253
HLA-B27:08	1306
HLA-B27:09	1363
HLA-B35:01	680
HLA-B35:03	23
HLA-B35:08	93
HLA-B37:01	39
HLA-B39:06	495
HLA-B40:01	1286
HLA-B40:02	1548
HLA-B41:01	19
HLA-B41:03	55
HLA-B41:04	37
HLA-B44:02	1662
HLA-B44:03	303
HLA-B44:27	24
HLA-B44:28	18
HLA-B45:01	150
HLA-B49:01	119
HLA-B50:01	114

HLA-B08:02	1018
HLA-B08:03	469
HLA-B14:01	42
HLA-B14:02	283
HLA-B15:01	4213
HLA-B15:02	164
HLA-B15:03	604
HLA-B15:09	830
HLA-B15:17	1444
HLA-B15:42	364
HLA-B18:01	2370
HLA-B27:01	4
HLA-B27:02	8
HLA-B27:03	874
HLA-B27:04	4
HLA-B27:05	3372
HLA-B27:06	7
HLA-B27:10	2
HLA-B27:20	92
HLA-B35:01	2724
HLA-B35:03	93
HLA-B35:08	1
HLA-B37:01	50
HLA-B38:01	500
HLA-B39:01	1785
HLA-B40:01	2984
HLA-B40:02	712
HLA-B40:13	59
HLA-B42:01	160
HLA-B42:02	18
HLA-B44:02	1954
HLA-B44:03	1006
HLA-B45:01	627
HLA-B45:06	362
HLA-B46:01	1798
HLA-B48:01	881
HLA-B51:01	2383
HLA-B52:01	12
HLA-B53:01	1341
HLA-B54:01	731
HLA-B57:01	2640
HLA-B57:02	18
HLA-B57:03	34
HLA-B58:01	3116
HLA-B58:02	56
HLA-B73:01	122
HLA-B81:01	26
HLA-B83:01	339
HLA-C03:03	153
HLA-C04:01	552
HLA-C05:01	172
HLA-C06:02	309
HLA-C07:01	241
HLA-C07:02	142

HLA-B51:01	2424
HLA-B57:01	370
HLA-C03:04	29
HLA-C04:01	366
HLA-C05:01	435
HLA-C07:02	19
HLA-C16:01	222
H2-Db	808
H2-Kb	1906
H2-Kd	663
Mamu-B008:01	495

HLA-C08:02	87
HLA-C12:03	172
HLA-C14:02	259
HLA-C15:02	252
HLA-E01:01	96
HLA-E01:03	55
Mamu-A01	2466
Mamu-A02	1188
Mamu-A07	535
Mamu-A11	1144
Mamu-A20102	132
Mamu-A2201	582
Mamu-A2601	142
Mamu-A70103	95
Mamu-B01	444
Mamu-B03	973
Mamu-B04	2
Mamu-B08	952
Mamu-B1001	140
Mamu-B17	1384
Mamu-B3901	439
Mamu-B52	846
Mamu-B6601	101
Mamu-B8301	368
Mamu-B8701	144
Patr-A0101	337
Patr-A0301	262
Patr-A0401	233
Patr-A0602	1
Patr-A0701	495
Patr-A0901	621
Patr-B0101	636
Patr-B0901	1
Patr-B1301	196
Patr-B1701	8
Patr-B2401	293
SLA-10401	185
SLA-10701	23
SLA-20401	105
SLA-30401	76
BoLA-AW10	166
BoLA-D18.4	258
BoLA-HD6	268
BoLA-JSP.1	158
BoLA-T2a	167
BoLA-T2b	157
BoLA-T2C	90
Gogo-B0101	14
H-2-Db	2580
H-2-Dd	276
H-2-Kb	3694
H-2-Kd	811
H-2-Kk	364
H-2-Ld	260

H-2-Lq	2

Supplementary Table 3. Multi Allele (MA) data summary for the HLA-II benchmark.

ID	Positives	Negatives	HLA-DRB		Source PMID
HLA-II-1	509	5355	DRB1*01:01	DRB1*04:01	27726376
HLA-II-2	240	2655	DRB1*01:01	DRB1*08:01	27726376
HLA-II-3	200	1890	DRB1*01:03	DRB1*03:01	27726376
HLA-II-4	327	3285	DRB1*03:01	DRB1*03:05	27726376
HLA-II-5	595	6930	DRB1*03:01	DRB1*15:01	27452731, 27726376
HLA-II-6	670	7740	DRB1*04:01	DRB1*07:01	27452731
HLA-II-7	1772	17460	DRB1*04:01	DRB1*10:01	27726376
HLA-II-8	213	2520	DRB1*04:01	DRB1*13:01	27452731
HLA-II-9	51	480	DRB1*04:01	DRB1*15:01	27726376
HLA-II-10	210	2475	DRB1*04:02	DRB1*11:04	27726376
HLA-II-11	682	7335	DRB1*04:03	DRB1*15:01	27726376
HLA-II-12	3216	29565	DRB1*07:01	DRB1*08:01	29632711
HLA-II-13	145	1710	DRB1*07:01	DRB1*13:01	27452731
HLA-II-14	496	4860	DRB1*08:01	DRB1*13:01	27452731, 29632711
HLA-II-15	121	1440	DRB1*08:01	DRB1*15:01	27726376
HLA-II-16	3080	29745	DRB1*08:03	DRB1*13:01	29632711
HLA-II-17	118	1215	DRB1*10:01	DRB1*11:01	27726376
HLA-II-18	257	2835	DRB1*11:01	DRB1*13:02	27726376
HLA-II-19	65	585	DRB1*13:01	DRB1*14:01	27452731
HLA-II-20	2426	22365	DRB1*15:01	DRB5*01:01	28467828

“Positives” and “Negatives” refer to the number of positive and negative instances contained in each MA EL data set. Further rows show the HLA-DRB alleles expressed by a given cell line, together with the Source PMID(s) for its corresponding dataset.

Supplementary Table 4. Multi Allele (MA) data summary for the BoLA benchmark.

<table>
<tr><th>Cell line ID</th><th>Haplotype</th><th>Positives</th><th>Negatives</th><th>BoLA-1</th><th>BoLA-2</th><th>BoLA-3</th><th>BoLA-4</th><th>BoLA-6</th><th>Source PMID</th></tr>
<tr><td rowspan="2">2123</td><td rowspan="2">A12/A15</td><td rowspan="2">11872</td><td rowspan="2">271523</td><td>BoLA-1*01901</td><td>BoLA-2*00801</td><td rowspan="2">-</td><td rowspan="2">BoLA-4*02401</td><td rowspan="2">-</td><td rowspan="6">-</td></tr>
<tr><td>BoLA-1*00901</td><td>BoLA-2*02501</td></tr>
<tr><td>5072</td><td>A11</td><td>8542</td><td>155590</td><td>-</td><td>BoLA-2*01801</td><td>BoLA-3*01701</td><td>-</td><td>-</td></tr>
<tr><td>2824</td><td>A19</td><td>9582</td><td>153620</td><td>-</td><td>BoLA-2*01601</td><td>-</td><td>-</td><td>BoLA-6*01402</td></tr>
<tr><td>5350</td><td>A20</td><td>11726</td><td>240196</td><td>-</td><td>BoLA-2*02601</td><td>BoLA-3*02701</td><td>-</td><td>-</td></tr>
<tr><td>2408</td><td>A15</td><td>24305</td><td>552309</td><td>BoLA-1*00901</td><td>BoLA-2*02501</td><td></td><td>BoLA-4*02401</td><td>-</td></tr>
<tr><td>1011/500004</td><td>A10</td><td>10188</td><td>148801</td><td>-</td><td>BoLA-2*01201</td><td>BoLA-3*00201</td><td>-</td><td>-</td><td rowspan="3">29115832</td></tr>
<tr><td>641</td><td>A18</td><td>6615</td><td>80170</td><td>-</td><td>-</td><td>-</td><td>-</td><td>BoLA-6*01301</td></tr>
<tr><td>2229/104003</td><td>A14</td><td>9509</td><td>186084</td><td>BoLA-1*02301</td><td>BoLA-2*02501</td><td>-</td><td>BoLA-4*02401</td><td>BoLA-6*04001</td></tr>
</table>

“Positives” and “Negatives” refer to the number of positive and negative instances contained in each cell line data. Further rows show the BoLA-1, BoLA-2, BoLA-3, BoLA-4, and BoLA-6 alleles expressed by a given cell line, together with the Source PMID for its corresponding dataset. Allele annotation was obtained from Vasoya, D. et al. (*64*)

Supplementary Table 5. Predicted Positive Predictive Values (PPV) for all clusters associated with each allele in each cell line the multi allele (MA) dataset after NNAlign_MA deconvolution. For details on the calculation of PPV, refer to materials and methods.

Cell Line ID	HLA Allele	PPV
Apher1	HLA-A03:01	0,84
	HLA-A29:02	0,73
	HLA-B44:02	0,81
	HLA-B44:03	0,91
	HLA-C12:03	0,75
	HLA-C16:01	0,49
Apher6	HLA-A02:01	0,74
	HLA-A03:01	0,82
	HLA-B07:02	0,91
	HLA-C07:02	0,38
Bcell	HLA-A01:01	0,79
	HLA-A03:01	0,75
	HLA-B07:02	0,88
	HLA-B27:05	0,83
	HLA-C02:02	0,53
	HLA-C07:01	0,39
CA46	HLA-A26:03	0,35
	HLA-B27:04	0,90
	HLA-C12:02	0,77
CD165	HLA-A02:05	0,64
	HLA-A24:02	0,81
	HLA-B15:01	0,87
	HLA-B50:01	0,82
	HLA-C03:03	0,75
	HLA-C06:02	0,83
CM467	HLA-A01:01	0,90
	HLA-A24:02	0,84
	HLA-B13:02	0,80
	HLA-B39:06	0,75
	HLA-C06:02	0,83
	HLA-C12:03	0,69
Fibroblast	HLA-A03:01	0,84
	HLA-A23:01	0,84
	HLA-B08:01	0,67
	HLA-B15:18	0,88
	HLA-C07:02	0,61
	HLA-C07:04	0,50
GD149	HLA-A01:01	0,79
	HLA-A24:02	0,78
	HLA-B38:01	0,90
	HLA-B44:03	0,92
	HLA-C06:02	0,82
	HLA-C12:03	0,54
HCC1143	HLA-A31:01	0,84
	HLA-B35:08	0,82
	HLA-B37:01	0,77

	HLA-C04:01	0,59
	HLA-C06:02	0,80
HCC1937	HLA-A23:01	0,55
	HLA-A24:02	0,87
	HLA-B07:02	0,83
	HLA-B40:01	0,90
	HLA-C03:04	0,78
	HLA-C07:02	0,35
HCT116	HLA-A01:01	0,86
	HLA-A02:01	0,74
	HLA-B18:01	0,86
	HLA-B45:01	0,89
	HLA-C05:01	0,71
	HLA-C07:01	0,59
HEK293	HLA-A03:01	0,90
	HLA-B07:02	0,71
	HLA-C07:02	0,54
HL-60	HLA-A01:01	0,38
	HLA-B57:01	0,92
	HLA-C06:02	0,81
JY	HLA-A02:01	0,85
	HLA-B07:02	0,91
	HLA-C07:02	0,48
LNT-229	HLA-A03:01	0,82
	HLA-B35:01	0,69
	HLA-C04:01	0,47
MAVER-1	HLA-A24:02	0,85
	HLA-A26:01	0,81
	HLA-B38:01	0,92
	HLA-B44:02	0,90
	HLA-C05:01	0,54
	HLA-C12:03	0,62
MD155	HLA-A02:01	0,76
	HLA-A24:02	0,84
	HLA-B15:01	0,88
	HLA-B18:01	0,87
	HLA-C03:03	0,72
	HLA-C07:01	0,45
Mel-12	HLA-A01:01	0,77
	HLA-B08:01	0,91
	HLA-C07:01	0,63
Mel-15	HLA-A03:01	0,76
	HLA-A68:01	0,81
	HLA-B27:05	0,76
	HLA-B35:03	0,82
	HLA-C02:02	0,48
	HLA-C04:01	0,38
Mel-16	HLA-A01:01	0,77
	HLA-A24:02	0,85
	HLA-B07:02	0,89
	HLA-B08:01	0,87
	HLA-C07:01	0,80
	HLA-C07:02	0,52

Mel-5	HLA-A01:01	0,52
	HLA-A25:01	0,82
	HLA-B08:01	0,82
	HLA-B18:01	0,87
Mel-624	HLA-A02:01	0,82
	HLA-A03:01	0,86
	HLA-B07:02	0,31
	HLA-B14:01	0,71
	HLA-C07:02	0,62
	HLA-C08:01	0,25
Mel-8	HLA-A01:01	0,84
	HLA-A03:01	0,79
	HLA-B07:02	0,90
	HLA-B08:01	0,84
	HLA-C07:01	0,75
	HLA-C07:02	0,54
PD42	HLA-A02:06	0,58
	HLA-A24:02	0,88
	HLA-B07:02	0,91
	HLA-B55:01	0,82
	HLA-C01:02	0,53
	HLA-C07:02	0,48
RA957	HLA-A02:20	0,64
	HLA-A68:01	0,83
	HLA-B35:03	0,87
	HLA-B39:01	0,89
	HLA-C04:01	0,72
	HLA-C07:02	0,53
RPMI8226	HLA-A30:01	0,66
	HLA-A68:02	0,79
	HLA-B15:03	0,77
	HLA-B15:10	0,91
	HLA-C02:10	0,60
	HLA-C03:04	0,73
SK-Mel-5	HLA-A02:01	0,81
	HLA-A11:01	0,85
	HLA-B40:01	0,86
	HLA-C03:03	0,57
T98G	HLA-A02:01	0,78
	HLA-B39:06	0,65
	HLA-C07:02	0,43
THP-1	HLA-A02:01	0,81
	HLA-A24:02	0,83
	HLA-B15:11	0,63
	HLA-B35:01	0,84
	HLA-C03:03	0,76
TIL1	HLA-A02:01	0,78
	HLA-B18:01	0,81
	HLA-B38:01	0,91
	HLA-C05:01	0,53
TIL3	HLA-A01:01	0,86
	HLA-A23:01	0,80
	HLA-B07:02	0,86
	HLA-B15:01	0,85

	HLA-C12:03	0,61
	HLA-C14:02	0,68
U-87	HLA-A02:01	0,78
	HLA-B44:02	0,81
	HLA-C05:01	0,65
pat-AC2	HLA-A03:01	0,79
	HLA-A32:01	0,79
	HLA-B27:05	0,78
	HLA-B45:01	0,84
pat-C	HLA-A02:01	0,51
	HLA-A03:01	0,58
	HLA-B07:02	0,78
	HLA-C07:02	0,65
pat-CELG	HLA-A02:01	0,60
	HLA-A24:02	0,82
	HLA-B15:01	0,80
	HLA-B73:01	0,70
	HLA-C03:03	0,61
	HLA-C15:05	0,61
pat-CP2	HLA-A11:01	0,87
	HLA-B14:02	0,80
	HLA-B44:02	0,93
pat-FL	HLA-A03:01	0,77
	HLA-A11:01	0,83
	HLA-B44:03	0,89
	HLA-B50:01	0,78
pat-J	HLA-A02:01	0,66
	HLA-A03:01	0,70
	HLA-B07:02	0,79
	HLA-C07:02	0,51
pat-JPB3	HLA-A02:01	0,85
	HLA-A11:01	0,82
	HLA-B27:05	0,83
	HLA-B56:01	0,72
pat-JT2	HLA-A11:01	0,89
	HLA-B18:03	0,74
	HLA-B35:01	0,78
pat-M	HLA-A03:01	0,63
	HLA-A29:02	0,61
	HLA-B08:01	0,68
	HLA-B44:03	0,86
	HLA-C07:01	0,44
	HLA-C16:01	0,59
pat-MA	HLA-A02:01	0,72
	HLA-A29:02	0,64
	HLA-B44:03	0,92
	HLA-B57:01	0,69
	HLA-C07:01	0,53
	HLA-C16:01	0,62
pat-ML	HLA-A02:01	0,70
	HLA-A11:01	0,77
	HLA-B40:01	0,89
	HLA-B44:03	0,93

pat-NS2	HLA-A02:01	0,87
	HLA-B13:02	0,74
	HLA-B41:01	0,71
pat-NT	HLA-A01:01	0,74
	HLA-A32:01	0,75
pat-PF1	HLA-A01:01	0,71
	HLA-A02:01	0,72
	HLA-B07:02	0,89
	HLA-B44:03	0,92
	HLA-C07:02	0,33
	HLA-C16:01	0,45
pat-R	HLA-A03:01	0,57
	HLA-A29:02	0,61
	HLA-B08:01	0,65
	HLA-B44:03	0,86
	HLA-C07:01	0,83
	HLA-C16:01	0,48
pat-RT	HLA-A01:01	0,69
	HLA-A02:01	0,71
	HLA-B18:01	0,85
	HLA-B39:24	0,79
	HLA-C05:01	0,54
	HLA-C07:01	0,44
pat-SR	HLA-A02:01	0,73
	HLA-A23:01	0,80
	HLA-B18:01	0,85
	HLA-B44:03	0,91
pat-ST	HLA-A03:01	0,82
	HLA-A24:02	0,86
	HLA-B07:02	0,89
	HLA-B27:05	0,84

Supplementary Table 6. Cell lines with atypical HLA-A or HLA-B peptidome repertoire profiles.

Cell line ID	HLA-A	HLA-B	HLA-C	Source PMID(s)
HL-60	0.047	0.851	0.102	26992070<br>17083564
CA46	0.071	0.739	0.191	26992070
Mel-624	0.931	0.048	0.021	7541714
HEK293	0.896	0.080	0.024	26258424

For each cell line, the relative peptidome size for the HLA-A, HLA-B and HLA-C loci is given. Peptidome sizes were calculated as described in Figure 3 B. The last column shows the references to earlier publications describing the loss of the locus for a given cell line.

Supplementary Table 7. AUC0.1 performance values for the SA molecules left out from the training of the A2 and A3 specificity reduced SA and MA models.

	SA MODEL	MA MODEL
HLA-A*02:01	0.376	0.778
HLA-A*02:05	0.533	0.771
HLA-A*02:07	0.477	0.871
HLA-A*03:01	0.359	0.896
HLA-A*11:01	0.361	0.862
HLA-A*26:01	0.838	0.962
HLA-A*29:01	0.762	0.873
HLA-A*31:01	0.409	0.749
HLA-A*32:01	0.808	0.905

Note that not all the molecules included in the evaluation are part of the A2 and A3 supertypes; these molecules are included because they have a distance to the A2 and A3 molecules in the MA dataset less than 0.1.
